# Nitrogen Fertilizers Technologies for Corn in Two Yield Environments in South Brazil

**DOI:** 10.3390/plants11141890

**Published:** 2022-07-21

**Authors:** Bruno Maia Abdo Rahmen Cassim, Marcos Renan Besen, Wagner Deckij Kachinski, Celso Rafael Macon, João Henrique Vieira de Almeida Junior, Rodrigo Sakurada, Tadeu Takeyoshi Inoue, Marcelo Augusto Batista

**Affiliations:** 1 Department of Agronomy, Maringá State University, Colombo Avenue, 5790, Zone 07, Maringá 87020900, Brazil; marcos.besen@hotmail.com (M.R.B.); wdkachinski@gmail.com (W.D.K.); ra105324@uem.br (C.R.M.); jvieiradealmeidajr@gmail.com (J.H.V.d.A.J.); ttinoue@uem.br (T.T.I.); 2 Cocamar Cooperativa Agroindustrial, Street Osvaldo de Moraes Corrêa, 1000, Maringá 87065590, Brazil; rodrigo.sakurada@cocamar.com.br

**Keywords:** urea, ammonia volatilization, enhanced-efficiency fertilizers, nitrification inhibitor, plant nutrition, X-ray diffraction, scanning electron microscopy

## Abstract

Improvements in nitrogen use efficiency (NUE) in corn production systems are necessary, to decrease the economic and environmental losses caused by loss of ammonia volatilization (NH_3_-N). The objective was to study different nitrogen (N) fertilizer technologies through characterization of N sources, NH_3_-N volatilization losses, and their effects on the nutrient concentration and yield of corn grown in clayey and sandy soils in south Brazil. The treatments consisted of a control without N application as a topdressing, three conventional N sources (urea, ammonium sulfate, and ammonium nitrate + calcium sulfate), and three enhanced-efficiency fertilizers [urea treated with NBPT + Duromide, urea formaldehyde, and polymer-coated urea (PCU) + urea treated with NBPT and nitrification inhibitor (NI)]. The losses by NH_3_-N volatilization were up to 46% of the N applied with urea. However, NI addition to urea increased the N losses by NH_3_-N volatilization by 8.8 and 23.3%, in relation to urea alone for clayey and sandy soils, respectively. Clayey soil was 38.4% more responsive than sandy soil to N fertilization. Ammonium sulfate and ammonium nitrate + calcium sulfate showed the best results, because it increased the corn yield in clayey soil and contributed to reductions in NH_3_-N emissions of 84 and 80% in relation to urea, respectively.

## 1. Introduction

Producing food sustainably and sufficiently for humanity has been a challenge over time. At the global level, corn (*Zea mays* L.) is the most produced grain, with 1.2 billion tons produced per year [1], and it will be responsible for a 45% increase in cereal production in the coming years [2], driven by an estimated population expansion to 9.7 billion people by 2050. Although the production potential of corn hybrids has increased through genetic improvements and the development of more technically advanced crops, the world average yield is 5980 kg ha^−1^ [1], far below the productive potential of the crop.

Nitrogen fertilization management is one of the factors that contributes the most to increasing corn yield. In plants, nitrogen (N) is the mineral element required in greatest quantities and is responsible for the synthesis of amino acids, proteins, and enzymes, and for photosynthetic processes [3]. Urea [CO(NH_2_)_2_] is the most commonly used source to meet the N needs of plants, because it has industrial advantages, such as a high N concentration per unit mass (45 to 46%) and lower production costs than other N sources [4]. It is estimated that in 2023, the global demand for N will be 155 Mt yr^−1^, of which 53% will be supplied by urea [5]. However, once applied to soil, urea is hydrolyzed by the action of urease enzyme, producing ammonia (NH_3_-N), which is rapidly lost to the atmosphere in the form of gas [6]. This loss may account for more than 60% of the N applied [7], depending on the soil and air temperature [8], soil moisture [9], soil pH [10], soil buffering capacity [11], presence of straw on the soil surface [12], N source [13], and rate of applied fertilizer N [14].

Although NH_3_-N is not a greenhouse gas (GHG), it can indirectly contribute to nitrous oxide (N_2_O-N) emissions [15], which are extremely harmful, due to their high global warming potential and permanence in the atmosphere for long periods [16]. Ammonia losses can reduce the N use efficiency (NUE), because less nutrients are left for plant absorption, causing negative yields and economic consequences for farmers [17,18]. In addition, NH_3_-N losses in agricultural areas affect air quality and contaminate terrestrial and aquatic ecosystems [19]. In the United States, for example, economic losses of approximately 39 billion dollars and deaths of more than 4300 people annually are linked to air pollution, as a result of NH_3_-N emissions from corn production systems that have both low NUE and nitrogen fertilizer overdoses [20].

The incorporation of urea into the soil is an effective way to reduce losses by NH_3_-N volatilization and increase NUE. This incorporation can be achieved using mechanical techniques [21] or irrigation [22]. Such practices are not always possible, because less than 20% of the world’s areas are irrigated [23] and because they interrupt the no-tillage system, which is an important soil conservation management practice. Therefore, surface application of N is the predominant practice in agricultural production systems. Nitrogen sources such as ammonium sulfate [(NH_4_)_2_SO_4_] and ammonium nitrate (NH_4_NO_3_) are not subject to considerable losses by NH_3_-N volatilization [13,24], but tend to be more expensive, due to their lower N concentration. In addition, ammonium nitrate is subject to purchase restrictions by the military, due to its use as an explosive material [4].

To circumvent these limitations, N fertilizer industries have relied on the use of urea as a raw material, due to its high concentration of N for the development of N enhanced-efficiency fertilizers (EEFs), classified as stabilized, slow-release, and controlled-release fertilizers [25]. However, although meta-analysis studies have revealed potential reduction in NH_3_-N losses by EEFs in relation to urea of between 39.4 and 52.0%, depending on the soil characteristics and climatic conditions before fertilizer application [26,27], the gains in crop yield are low compared to those obtained with conventional urea, ranging from 5.3 to 6.0% [4,26,27].

The N fertilizer industry has developed new stabilizing molecules to inhibit urease activity and has proposed combinations of enhanced-efficiency technologies, to obtain mixed (two or more granules) and/or complex (single-granule) fertilizers. For example, the new Duromide + N-(n-butyl) thiophosphoric triamide (NBPT) stabilization technology reduced NH_3_-N losses by 33% compared to only NBPT [9]. On the other hand, the addition of nitrification inhibitors (NIs), which aims to reduce N_2_O-N losses and nitrate leaching (NO_3_^−^-N) [28,29], increases the volatilization of NH_3_-N by 35.7% and consequently the indirect emissions of N_2_O-N by up to 15.2% [30], leading to major debates on the use of NIs to increase NUE in EEFs [31].

Therefore, the objective of this work was to study the different technologies of N fertilizers through the characterization of their N sources, NH_3_-N volatilization losses, and effects on the nutrient concentration and yield of corn grown in clayey and sandy soil in south Brazil.

## 2. Materials and Methods

### 2.1. Description of the Sites and Soils

**Figure 1:**
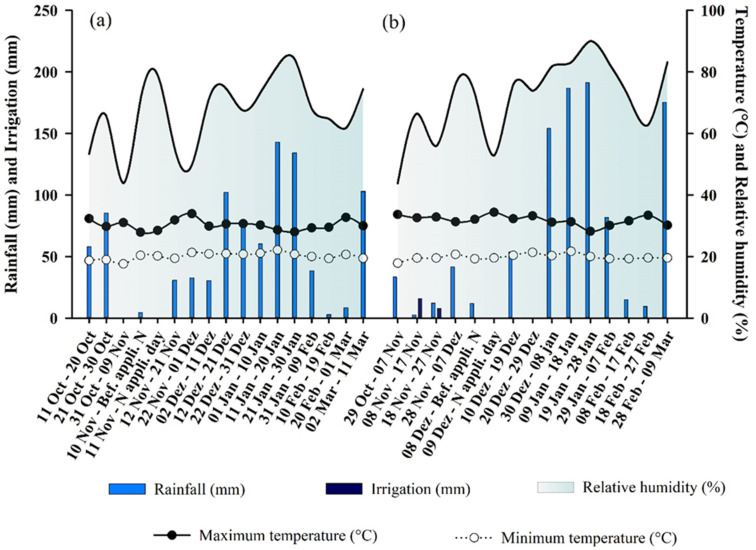
Pluviometric precipitation (mm), air relative humidity (%), maximum and minimum temperature (°C), and irrigation (mm) for corn grown on clayey soil (**a**) and corn grown on sandy soil (**b**). Bef. appli. N: Before application nitrogen day in topdressing. N appli. Day: Day of nitrogen application in topdressing.

The experiments were conducted in two locations belonging to the Technology Diffusion Unit (UDT) of Cocamar Cooperativa Agroindustrial: one located in the municipality of Floresta (23°35′37″ S; 52°04′06″ W), and another in the municipality of Guairaçá (22°56′48″ S; 52°43′22″ W), at 392 and 478 m above sea level, respectively. The climate of the study area is classified as subtropical humid mesothermal (Cfa) according to the Köppen-Geiger classification system [32]. The rainfall, temperature, relative air humidity, and irrigation depth data during the experiments are shown in Figure 1.

The experiments were conducted in the municipalities of Floresta and Guairaçá, located in the state of Paraná, Brazil, in no-till areas, with previous crops of *Brachiaria ruziziensis* and *B. brizantha*, respectively. The soils of the experimental areas in Floresta and Guairaçá were classified as a Latossolo Vermelho distroférrico with clayey texture (clayey soil) and Argissolo Vermelho-Amarelo distrófico (sandy soil) [33], corresponding to an Oxisol and Ultisol, respectively, according to the soil taxonomy of the USDA [34]. Soil samples from the 0–20 m layer were collected for chemical characterization and the determination of particle size (Table 1).

### 2.2. Experimental Design, Treatments, and Crop Management

A randomized block experimental design was applied, with five replicates and seven treatments. The treatments consisted of a control without N application as topdressing; three conventional nitrogen sources: urea (46% N), ammonium sulfate (21% N and 24% S), and ammonium nitrate + calcium sulfate (27% N, 3.7% S and 5% Ca); and three fertilizers with increased efficiency: one fertilizer stabilized to inhibit the activity of urease enzyme [urea treated with NBPT + Duromide (46% N)], one slow-release fertilizer [urea formaldehyde (37% N)], and one fertilizer consisting of a mixture of granules of different technologies [polymer-coated urea (PCU) (42% N) + urea treated with NBPT and nitrification inhibitor (Ur-NBPT + NI) (46% N) + 3.0% S and 0.3% B in the form of elemental sulfur (99% S) and ulexite (10% B), respectively]. The experimental units were 4 m wide and 10 m long, yielding a total area of 40 m^2^.

Corn (*Zea mays* L.) was sown at the Floresta (clayey soil) and Guairaçá (sandy soil) sites on 14 October and 6 November 2020, under a dry mass cover of 3.15 and 4.76 Mg ha^−1^ *B. ruziziensis* and *B. brizantha*, respectively. The corn hybrids used in Floresta and Guairaçá were Brevant 2433 PWU and FS512 PWU, respectively, with a distribution of 2.7 seeds m^−1^ and a spacing of 0.45 m, totaling 60,000 pl ha^−1^. Sowing fertilization was performed with the application of 535 and 400 kg ha^−1^ of 10-15-15 (N-P_2_O_5_-K_2_O), and when the corn was at phenological stage V4 (four leaves with collars visible), 60 and 40 kg ha^−1^ K_2_O as KCl were applied in Floresta and Guairaçá, respectively. The N fertilizers were applied as a topdressing, according to the expected yield of the Floresta (clayey soil) and Guairaçá (sandy soil) sites at phenological stage V5 (five leaves with collars visible) at doses of 200 and 150 kg ha^−1^, respectively, as recommended by the Parana State Fertilization and Liming Manual (SBCS/NEPAR) [35]. 

### 2.3. Capture and Determination of Ammonia Volatilization

To determine the ammonia volatilization, the N fertilizers were weighed separately with an analytical balance and manually applied in a semi-open static chamber allocated within each experimental unit. For the treatment consisting of a mixture of granules of different technologies, PCU was physically separated from Ur-NBPT + NI, and three chambers were installed in the experimental unit for each treatment; with the first chamber for PCU, the second chamber for Ur-NBPT + NI, and the third chamber for granules mixed at a ratio of 50% PCU and 44% Ur-NBPT + NI, as the product is marketed.

Immediately after the application of N fertilizers in Floresta (clayey soil) and Guairaçá (sandy soil), N losses via ammonia volatilization were quantified through sample collection, performed at 1, 2, 4, 6, 8, 11, 15, 19, 22, 26, 33, 40, 47, 54, 61, 68, 76, and 83 days and 1, 2, 4, 6, 9, 15, 21, 28, 36, 44, 51, 58, 65, 71, and 78 days after application, totaling 18 and 15 collection times, respectively. The chambers were constructed from plastic bottles (polyethylene terephthalate, PET) with a total area of 0.007854 m^2^. Each chamber contained a 2.5 cm wide and 25 cm long strip of filter paper with a base immersed in a 50-cm^3^ flask with 20 mL 0.05 mol L^−1^ H_2_SO_4_ and a solution of 2% glycerine (*v*/*v*) [36,37]. The used vials were replaced with new vials until the ammonia loss stabilized.

After each collection, the chambers were rotated between the three bases within each experimental unit, to minimize the effects of environmental factors such as rainfall and temperature. Subsequently, the samples were sent to the Soil Fertility Laboratory of the Maringá State University, Paraná, Brazil, and refrigerated until analysis. Ammonia captured in the form of ammonium sulfate was determined by UV/VIS spectrophotometry, using the salicylate green method [37]. During the experimental period of ammonia volatilization sampling, no irrigation was performed.

### 2.4. Characterization of Nitrogen Fertilizers

The N fertilizers were finely ground and characterized by X-ray diffraction (XRD) analysis (XRD 6000, Shimadzu, Kyoto, Japan). X-ray diffractograms were obtained with a scanning interval of 3° to 70° 2θ, sampling step of 0.02°, and time of 1.2 s using CoKα radiation with a nickel filter (40 kV, 30 mA). The values obtained were exported to X’Pert Highcore Plus software to determine the intensity, peak position, and crystallographic *hkl* plane. To identify the coating layer and coating thickness of the PCU + Ur-NBPT + NI treatment with controlled-release technology, the granules were physically separated into PCU and Ur-NBPT + NI and cut longitudinally with the aid of a scalpel blade. Subsequently, the granules were fixed to a stub microscope support with the aid of carbon tape and then metallized with gold. The samples were then analyzed using scanning electron microscopy (SEM), using a Quanta FEG 250 microscope.

### 2.5. Evaluation of Nutrient Concentration, Morphological, and Yield Status

During the flowering period of the corn, corresponding to phenological stage R1 (silking), indirect readings of the chlorophyll leaf content (SPAD index) were performed using SPAD 502 Plus^®^ instrument (Konica Minolta, Tokyo, Japan). The nutrient concentration of the corn was evaluated at phenological stage R1, by randomly collecting 15 plants from the middle third of the first leaf opposite and below the upper ear [35]. After collecting the leaves, the samples were sent to the Soil Fertility Laboratory of the Maringá State University, Paraná, Brazil; washed with distilled water; dried in an oven with forced air circulation at 65 °C for 72 h; and ground in a Wiley mill. Subsequently, the samples were weighed and subjected to sulfuric acid and nitric-perchloric acid digestion for the extraction of N, phosphorus (P), potassium (K), calcium (Ca), magnesium (Mg), sulfur (S), zinc (Zn), copper (Cu), iron (Fe), and manganese (Mn). For analysis of leaf boron (B) content, the samples were subjected to dry digestion via calcination in a muffle furnace [38].

The content of Ca, Mg, Zn, Cu, Fe, and Mn were determined by atomic absorption spectrophotometry (AAS) with a mixture of air:acetylene. Phosphorus was determined by vanadate yellow spectrophotometry, S by spectrophotometry using the barium sulfate turbidimetry method, K by flame emission photometry, N by the micro-Kjeldahl method, and B by azomethine-H spectrophotometry [38].

The plant height was determined at phenological stage R2 (milky grains) by measuring from the soil surface to the insertion of the tassel using a tape measure. After the physiological maturation of corn, corresponding to phenological stage R6, manual harvesting of the ears was performed in a useful area of 10.8 m^2^, the kernel moisture was corrected to 13%, and the kernel mass of each experimental unit was extrapolated to obtain the yield in kg ha^−1^.

### 2.6. Statistical Analysis

The data obtained for corn yield, height, and nutrient concentration were subjected to homogeneity of variance (Bartlett) and error normality (Lilliefors) tests, thus meeting the assumptions of analysis of variance [39]. Subsequently, the data were subjected to joint analysis of variance, provided that the quotient between the largest and smallest residual mean squares of the analysis of individual variances was less than 7 [40]. Treatments and places were considered fixed factors, and their interaction was subdivided into treatments within places and places within treatments (*p* < 0.05), as shown by the follow statistical model. Subsequently, the means were compared using Tukey’s test at 5% probability, using the statistical software GENES [41].

Y*ijk* = *µ* + G*i* + B/A*jk* + A*j* + GA*ij* + ε*ijk*

where Y*ijk* is the observed value for treatment *i* (nitrogen fertilizer) in place *j* (clayey or sandy soil) in block *k*; *µ* is the effect of the mean; G*i* is the fixed effect of treatment i; B/A*jk* is the block nested in place *j*; A*j* is the fixed effect of place *j*; GA*ij* is the interaction between treatment *i* and place *j*; and ε*ijk* is the experimental random error in treatment *i*, place *j*, and block *k*.

For the NH_3_-N volatilization data, model selection was performed according to the Akaike information criterion (AIC) [42], and the model with the lowest AIC was chosen. After selecting the model, the data were subjected to nonlinear regression using SigmaPlot software, using the logistic model of three parameters (α, β and γ) represented by Equation (1), as described by Seber and Wild [43]. This model has traditionally been used to estimate plant growth and nutrient uptake rates [44] and has been more recently used to estimate losses by NH_3_-N volatilization [9,13].

(1) $$\hat{Y}=\frac{\alpha }{1+{\exp\;}^{\left[-\left(\mathrm{time}-\beta \right)/\gamma \right]}}\;$$

where $\hat{Y}$ is the amount of N volatilized as NH_3_-N (kg ha^−1^) at time t; α is the maximum accumulated volatilization; β is the time at which a 50% loss occurs, corresponding to the inflection point of the curve (the day when the maximum daily loss of NH_3_-N occurs); t is the time (days); and γ is a parameter of the model used to calculate the maximum daily loss (MDL) of NH_3_-N, as shown in Equation (2).

(2) $$\mathrm{MDL}=\frac{\alpha }{4\gamma }\;$$

## 3. Results

### 3.1. Scanning Electron Microscopy and X-ray Diffraction

**Figure 2:**
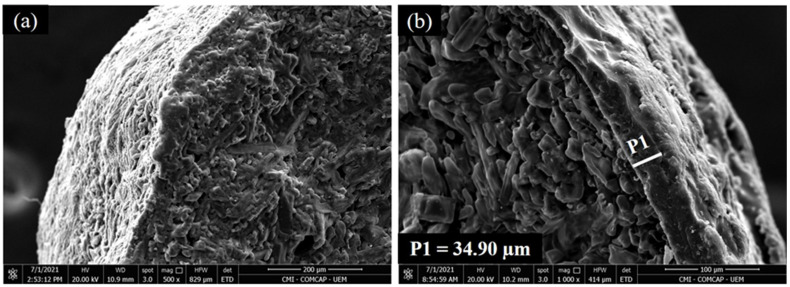
Electron micrographs of the separate Ur-NBPT + NI (**a**) and PCU granules (**b**). P1 is the thickness of the coating layer of the polymer coated granule.

The electron micrographs revealed the transverse morphology of the granules that compose the PCU + Ur-NBPT + NI granule mixture, indicating the presence or absence of coating (Figure 2). Thus, the Ur-NBPT + NI granules do not have a coating layer (Figure 2a), whereas the PCU granules have a polymer coating layer with an average thickness of 34.90 μm (Figure 2b).

**Figure 3:**
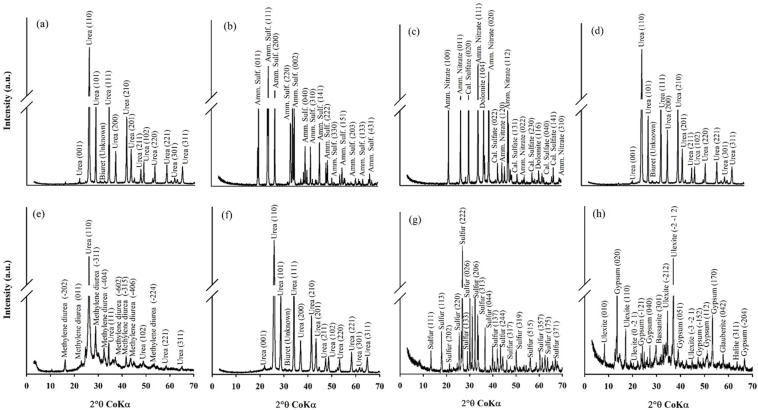
X-ray diffraction of the urea (Ur) (**a**), ammonium sulfate (**b**), ammonium nitrate + calcium sulfate (**c**), Ur-NBPT + Duromide (**d**), Ur-formaldehyde (**e**), PCU + Ur-NBPT + NI (**f**), elemental sulfur (**g**) and ulexite (**h**) for conventional and enhanced efficiency nitrogen fertilizers characterization.

The X-ray diffractograms showed typical reflections of the chemical species of each nitrogen fertilizer (Figure 3). The urea-based fertilizers (urea and Ur-NBPT + Duromide) showed characteristic reflections of urea (110) and biuret (Figure 3a,d). The only phase found in ammonium sulfate was the characteristic reflection of this fertilizer (Figure 3b). Conversely, the ammonium nitrate + calcium sulfate-based fertilizer contained dolomite, ammonium nitrate, and calcium sulfate, with more intense *hkl* planes at 104, 111, and 020, respectively (Figure 3c). In Ur-formaldehyde, at least two phases were identified, urea and methylenediurea (MDU), indicated by more intense reflections at 110 and −311, respectively (Figure 3e). 

For the mixed fertilizer, the granules were separated into PCU, Ur-NBPT + NI, S granules (elemental sulfur), and B granules (ulexite). The XRD patterns of PCU and Ur-NBPT + NI indicated reflections characteristic of urea and biuret (Figure 3f). The S granule was identified as elemental sulfur with a more intense plane at 222 (Figure 3g). The B granule showed several phases, such as ulexite, gypsum, glauberite halite, and bassanite, with more intense planes at -2-12, 020, 311, 042, and 301, respectively (Figure 3h).

### 3.2. N Losses through Ammonia Volatilization

**Figure 4:**
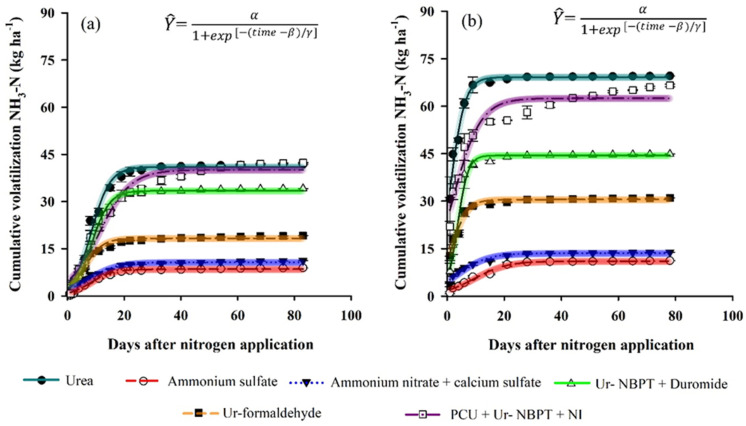
Cumulative volatilization of NH3-N after topdressing applications of urea, ammonium sulfate, ammonium nitrate + calcium sulfate, Ur-NBPT + Duromide, Ur-formaldehyde, and PCU + Ur-NBPT + NI for clayey soil at a rate of 200 kg ha^−1^ of N (**a**) and for sand soil at a rate of 150 kg ha^−1^ of N (**b**). Data with overlapping vertical bars with 95% confidence interval in the curve.

The climatic conditions before the application of the N topdressing fertilizers are shown in Figure 1. The fertilizers were applied 24 h after 4.6 and 12 mm rainfall to the clayey and sandy soil, respectively. The maximum and minimum temperatures during the first 76 h after fertilization were 31.1 and 19.9 °C, respectively, for Floresta (clayey soil) and 34.0 and 20.2 °C for Guairaçá (sandy soil). The volatilization of NH_3_-N followed a sigmoidal pattern, increasing at the beginning, reaching the maximum daily loss, and then stabilizing (Figure 4). The maximum accumulated loss (α) of NH_3_-N according to the adjusted model decreased in the following order: urea (41.0 and 69.2 kg ha^−1^ NH_3_-N; 20.5 and 46.1% of the N applied), PCU + Ur-NBPT + NI (40.1 and 62.5 kg ha^−1^ NH_3_-N; 20.0 and 41.7% of the N applied); Ur-NBPT + Duromide (33.4 and 44.5 kg ha^−1^ NH_3_-N; 16.7 and 29.7% of the N applied), Ur-formaldehyde (18.3 and 30.5 kg ha^−1^ NH_3_-N; 9.1 and 20.3% of the N applied), ammonium nitrate + calcium sulfate (10.7 and 13.6 kg ha^−1^ NH_3_-N; 5.3 and 9.1% of the N applied), and ammonium sulfate (8.5 and 11.0 kg ha^−1^ NH_3_-N; 4.2 and 7.3% of the N applied) for clayey and sandy soils, respectively (Table 2).

Ammonium sulfate, ammonium nitrate + calcium sulfate, Ur-formaldehyde, Ur-NBPT + Duromide, and PCU + Ur-NBPT + NI reduced NH_3_-N losses by 79.3 and 84.1, 73.9 and 80.3, 55.4 and 55.9, 18.5 and 35.7, and 2.2 and 9.7% compared to urea for clayey and sandy soil, respectively (Table 2). Ammonium sulfate and ammonium nitrate + calcium sulfate were the sources that most reduced the NH_3_-N volatilization losses in both locations compared to urea, with average reductions of 81.7 and 77.1%, respectively. The peak NH_3_-N volatilization (β) of urea, ammonium sulfate, ammonium nitrate + calcium sulfate, Ur-NBPT + Duromide, Ur-formaldehyde, and PCU + Ur-NBPT + NI occurred at 8.4 and 1.2, 8.3 and 7.1, 7.3 and 3.5, 8.5 and 3.6, 6.0 and 1.6, and 11.1 and 2.2 days after application of topdressing fertilizers to clayey and sandy soil, respectively (Table 2).

The environment in the sandy soil area provided lower β compared to the environment in the clayey soil area, and the NH_3_-N volatilization peaks advanced by 8.9, 7.2, 4.9, 4.4, 3.8 and 1.2 days for PCU + Ur-NBPT + NI, urea, Ur-NBPT + Duromide, Ur-formaldehyde, ammonium nitrate + calcium sulfate, and ammonium sulfate, respectively. The maximum daily loss (MDL) of NH_3_-N decreased in the following order: urea (3.10 and 6.92 kg ha^−1^ NH_3_-N), Ur -NBPT + Duromide (2.69 and 6.18 kg ha^−1^ NH_3_-N), PCU + Ur-NBPT + NI (1.82 and 3.32 kg ha^−1^ NH_3_-N), Ur-formaldehyde (1.43 and 2.82 kg ha^−1^ NH_3_-N), ammonium nitrate + calcium sulfate (0.54 and 0.64 kg ha^−1^ NH_3_-N), and ammonium sulfate (0.56 and 0.47 kg ha^−1^ NH_3_-N) for clayey and sandy soil, respectively (Table 2). The greatest reductions in MDL were obtained with the use of ammonium sulfate and ammonium nitrate + calcium sulfate in both locations.

### 3.3. Ammonia Volatilization in Granules with and without Nitrification Inhibitor

**Figure 5:**
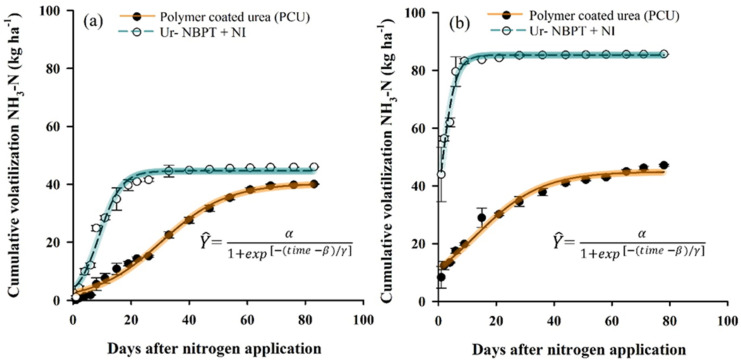
Cumulative volatilization of NH3-N after topdressing applications of the separate granules PCU and Ur-NBPT + NI for clayey soil at a rate of 200 kg ha^−1^ of N (**a**) and for sand soil at a rate of 150 kg ha^−1^ of N (**b**). Data with overlapping vertical bars with 95% confidence interval in the curve.

According to the fitted model (Figure 5), the maximum accumulated losses of NH_3_-N for the granules decreased in the order Ur -NBPT + NI (44.6 and 85.3 kg ha^−1^ NH_3_-N; 22.3 and 56.9% of the N applied) followed by PCU (40.3 and 44.9 kg ha^−1^ NH_3_-N; 20.1 and 29.9% of the N applied) for clayey and sandy soil, respectively (Table 3). Urea-NBPT + NI granules increased the NH_3_-N volatilization losses by 8.8 and 23.3% compared to urea for clayey and sandy soils, respectively.

The time of peak NH_3_-N volatilization of the granules occurred in the order PCU (30.6 and 12.6 days) followed by Ur-NBPT + NI (9.0 and 0.7 days), corresponding to delays in the peak of NH_3_-N volatilization of 21.6 and 11.9 days with PCU compared to Ur-NBPT + NI for clayey and sandy soil, respectively (Table 3). The MDL of Ur-NBPT + NI was 2.93 and 8.53 kg ha^−1^ NH_3_-N, followed by PCU with 0.89 and 0.98 kg ha^−1^ NH_3_-N for clayey and sandy soil, respectively (Table 3). The fertilizer Ur-NBPT + NI increased MDL by 69.6 and 88.5% relative to PCU for clayey and sandy soil, respectively.

### 3.4. Leaf Macronutrient Content in Corn

**Figure 6:**
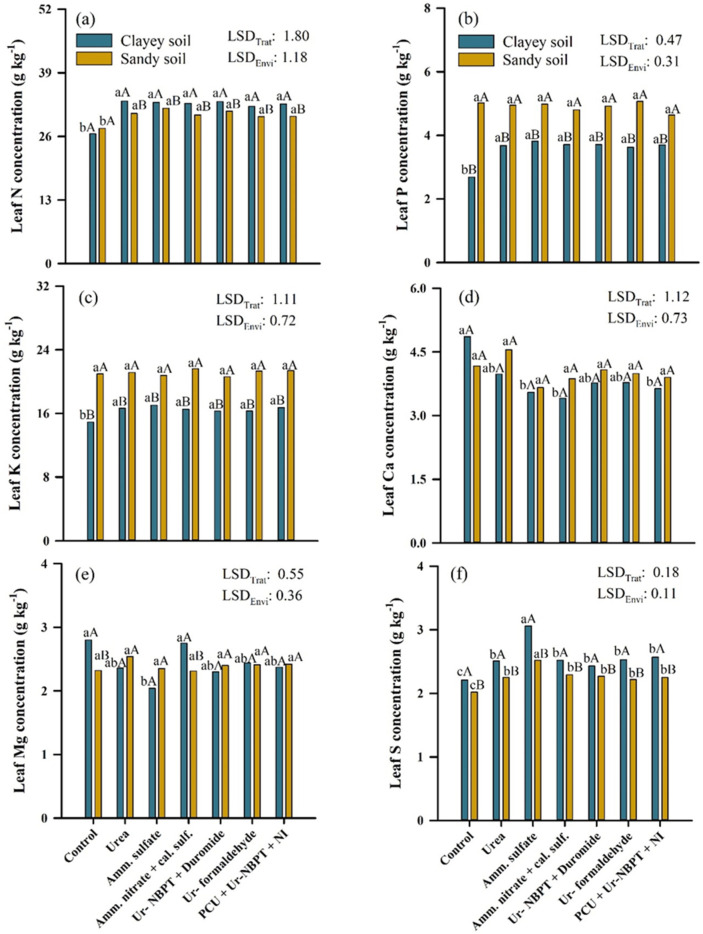
Concentration of nitrogen (**a**), phosphorus (**b**), potassium (**c**), calcium (**d**), magnesium (**e**), and sulfur (**f**) in the corn leaf after fertilization in topdressing with nitrogen sources urea, ammonium sulfate, ammonium nitrate + calcium sulfate, Ur-NBPT + Duromide, Ur-formaldehyde, and PCU + Ur-NBPT + NI for clayey and sand soil. Treatments followed by the same lowercase letter do not differ using a Tukey test (*p* < 0.05). Environments followed by the same capital letter do not differ using a Tukey test (*p* < 0.05). LSD_Trat_: least significant difference for treatments. LSD_Envi_: least significant difference for environment.

Regarding the leaf contents of macronutrients (Figure 6), the application of nitrogen fertilizers in topdressing increased leaf N contents by 6.31 (23.6%) and 3.05 (11.0%) g kg^−1^ in comparison to the control for the clayey and sandy soils, respectively. However, there was no difference between the nitrogen sources in either soils. The results for nitrogen fertilizers applied as a topdressing in clayey soil were 2.12 (6.9%) g kg^−1^ higher than those for the same applications performed in sandy soil, with the exception of the control, which showed no differences in leaf N content between the clayey and sandy soils (Figure 6a). The nitrogen sources increased the levels of leaf P and K only in a clayey soil environment, with a mean increase in relation to the control of 1.02 (37.9%) and 1.68 (11.7%) g kg^−1^, respectively, and no differences between the sources. All treatments, with or without application of N in topdressing, conducted in sandy soil had higher levels of leaf P and K than those conducted in clayey soil, with a mean difference of 1.35 (37.9%) and 4.78 (29.2%) g kg^−1^, respectively (Figure 6b,c).

In the clayey soil, corn without N fertilization as a topdressing had a higher leaf Ca content than the PCU + Ur-NBPT + NI, ammonium sulfate, and ammonium nitrate + calcium sulfate treatments, with increases of 1.22 (33.5%), 1.31 (36.9%), and 1.45 (42.5%) g kg^−1^, respectively. There was no significant difference between treatments for leaf Ca levels in corn grown in sandy soil and no significant differences between locations (Figure 6d). Regarding the levels of leaf Mg, in clayey soil, ammonium nitrate + calcium sulfate and the control had higher levels than ammonium sulfate, with differences of 0.76 (34.8%) and 0.71 (37.3%) g kg^−1^, respectively. There was no significant difference between treatments regarding leaf Mg content in the corn grown in sandy soil. Regarding the locations, the values for ammonium nitrate + calcium sulfate and the control in clayey soil were 0.44 (19.0%) and 0.48 (20.7%) g kg^−1^ higher than the corresponding values in sandy soil, respectively (Figure 6e).

The ammonium sulfate source provided the highest leaf S levels in corn in both clayey and sandy soil, with an average increase of 0.55 (21.9%) and 0.85 (38.5%) g kg^−1^ for clayey soil and 0.27 (12.0%) and 0.50 (24.7%) g kg^−1^ for sandy soil compared to the other nitrogen sources and the control, respectively. Although ammonium sulfate provided the largest increases in leaf S, the sources urea, ammonium nitrate, Ur-NBPT + Duromide, Ur-formaldehyde, and PCU + Ur-NBPT + NI also increased the levels of leaf S, but only in relation to the control, with average values of 0.30 (13.6%) and 0.23 (24.7%) g kg^−1^ for clayey and sandy soils, respectively. Corn, with or without application of N as a topdressing, grown in clayey soil had, on average, 0.29 (12.8%) g kg^−1^ more leaf S than corn grown in sandy soil (Figure 6f).

### 3.5. Leaf Micronutrient Content and SPAD Index

**Figure 7:**
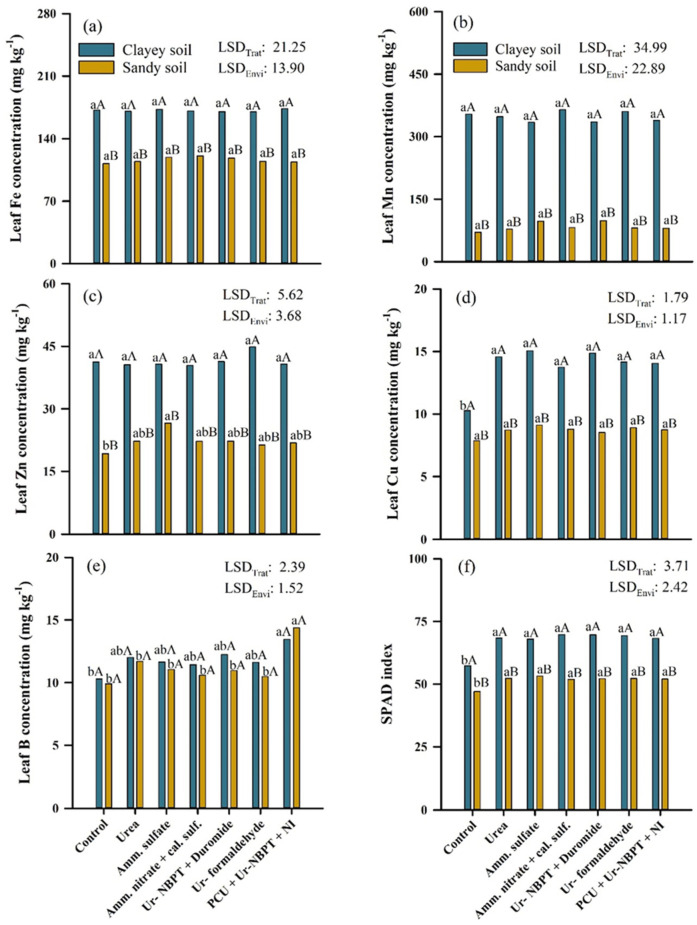
Concentration of iron (**a**), copper (**b**), zinc (**c**), manganese (**d**), boron (**e**), and SPAD index (**f**) in the corn leaf after fertilization in topdressing with nitrogen sources urea, ammonium sulfate, ammonium nitrate + calcium sulfate, Ur-NBPT + Duromide, Ur-formaldehyde, and PCU + Ur-NBPT + NI for clayey and sand soil. Treatments followed by the same lowercase letter do not differ using a Tukey test (*p* < 0.05). Environments followed by the same capital letter do not differ using a Tukey test (*p* < 0.05). LSD_Trat_: least significant difference for treatments. LSD_Envi_: least significant difference for environment.

Regarding the micronutrient contents (Figure 7), leaf Fe and Mn were not influenced by N fertilization as a topdressing for either the clayey or sandy soil. However, the levels of leaf Fe and Mn in corn cultivated in a clayey soil environment were higher, at 55.32 (47.6%) and 263.99 (313.9%) mg kg^−1^, than the values for corn cultivated in sandy soil, respectively (Figure 7a,b). For leaf Zn, there was no difference between treatments in clayey soil. However, in the ammonium sulfate treatment in sandy soil, the leaf Zn content increased in relation to the control by 7.34 (38.1%) mg kg^−1^. All treatments, with or without application of N as a topdressing, conducted in clayey soil were higher to those conducted in sandy soil, with a mean difference of 19.46 (88.5%) mg kg^−1^ in leaf Zn (Figure 7c). 

Leaf Cu levels increased with topdressing N fertilization only in relation to the control, with an average increase of 4.13 (40.1%) mg kg^−1^ for clayey soil; but in sandy soil, there was no difference between treatments. Similarly to Fe, Mn, and Zn, the levels of leaf Cu in corn grown in a clayey soil environment were also higher than those in corn grown in a sandy soil environment, with a mean difference of 5.16 (59.5%) mg kg^−1^ in leaf Cu (Figure 7d). The application of PCU + Ur -NBPT + NI increased the leaf B content in both the clayey and sandy environments; but in clayey soil, an increase of 3.18 (30.9%) mg kg^−1^ of B occurred only in relation to the control, while in the sandy soil, an increase of 3.41 (32.9%) and 4.46 (45.0%) mg kg^−1^ in leaf B occurred in relation to the other nitrogen sources and the control, respectively. There were no significant differences in leaf B content between clayey and sandy soil (Figure 7e).

Regarding the indirect chlorophyll content (SPAD), the application of N fertilizers as topdressing increased the SPAD index by 11.58 (20.2%) and 5.27 (11.2%) compared to the control for clayey and sandy soil, respectively. However, there was no difference between the nitrogen sources in either production environment. The SPAD indexes of all treatments, with or without application of N as topdressing, conducted in clayey soil were higher than those in sandy soil, with a mean difference of 15.67 (30.4%) (Figure 7f).

### 3.6. Yield and Height of Corn Plants

**Figure 8:**
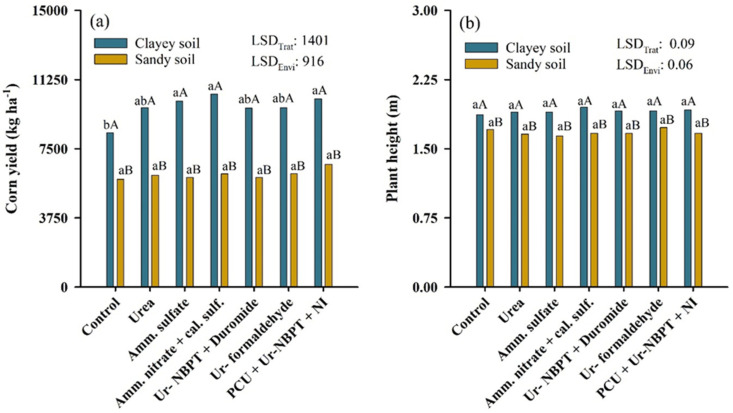
Corn yield (**a**) and plant height (**b**) after fertilization in topdressing with nitrogen sources urea, ammonium sulfate, ammonium nitrate + calcium sulfate, Ur-NBPT + Duromide, Ur-formaldehyde, and PCU + Ur-NBPT + NI for clayey and sand soil. Treatments followed by the same lowercase letter do not differ using a Tukey test (*p* < 0.05). Environments followed by the same capital letter do not differ using a Tukey test (*p* < 0.05). LSD_Trat_: least significant difference for treatments. LSD_Envi_: least significant difference for environment.

Nitrogen fertilizers increased the corn yield only in a clayey soil environment. Increases in yield were obtained with the use of ammonium sulfate, PCU + Ur-NBPT + NI and ammonium nitrate + calcium sulfate, which resulted in increases of 1722 (20.6%), 1838 (21.9%), and 2088 (24.9%) kg ha^−1^ corn, respectively, compared to the control that did not receive N fertilization as a topdressing. All treatments, with or without application of N as topdressing, conducted in clayey soil were higher than those conducted in sandy soil, with a mean difference in yield of 3654 kg ha^−1^, equivalent to 37.4%. However, when considering only the treatments that received N as a topdressing, the difference in yield became 370 kg ha^−1^, equivalent to 38.4% (Figure 8a). Topdressing N fertilization did not influence plant height in either the clayey or sandy soil environment. However, the plant height in clayey soil was 23 cm higher on average than that in sandy soil (Figure 8b).

## 4. Discussion

### 4.1. X-ray Diffraction and SEM of N Fertilizers

The industrial production of N fertilizers in amidic form (Figure 3a,d,f) produces biuret as a by-product, resulting from the increase in temperature above the melting point of urea, which is 132 °C [45]. Although biuret is a common impurity, Brazilian legislation allows only up to 2% in solid N fertilizer [46], because it is a toxic chemical compound that interferes with the protein synthesis of plants [47]. Currently, biuret toxicity is insignificant in crops, due to advances in the technology used to manufacture urea fertilizers [48]. Although Ur-formaldehyde comes from an amidic source, biuret was not found in the XRD analysis, probably due to strict controls in the production process.

Dolomite, which was identified in the X-ray diffractogram of ammonium nitrate + calcium sulfate (Figure 3c), inhibits the exothermic and undesirable decomposition of ammonium nitrate, thus improving the safety of the fertilizer [49]. According to standard NFPA 490 of the National Fire Protection Association [50], ammonium nitrate is not considered flammable or combustible. However, factors such as a high temperatures under confinement (260 to 300 °C) and contamination by organic or inorganic materials, such as chlorides or powdered metals, can lead to explosive detonation with the production of nitrous oxide, which is decomposed into nitrogen and oxygen [51,52].

Urea-formaldehyde was the first synthetic nitrogen fertilizer with low solubility to be marketed for slow release of N. The production process consists of condensation within a reactor with controlled pH, temperature, molar ratio, and reaction time between urea and formaldehyde [25]. The final product of the reaction consists of a mixture of methylene urea polymers (methylene urea, MDU and polymethylene) with differences in the degree of polymerization (insolubility) and molecular weight (chain length) [53,54]. Thus, the presence of MDU in Ur-formaldehyde (Figure 3e) will provide an intermediate molecular weight and degree of polymerization, contributing to the slow release of N. This compound, combined with certain amounts of unreacted urea, results in an intelligent fertilizer for agricultural use within the cultivation time of the plants. For example, Cassim et al. [55] observed no increase in yield in cultures with the application of Ur-formaldehyde with 70% slow-release compounds; but, with proportions of 55 and 60% of slow-release compounds, the yield increases were significant.

The lack of sulfur in the mixture of PCU + Ur-NBPT + NI granules (Figure 3f) indicates that the coating layer of the PCU granules (Figure 2b) is covered only by polymer and not by elemental sulfur (S^0^). This configuration provides better nutrient release kinetics than an S^0^ coating, because it is independent of the activity of microorganisms responsible for the oxidation of S^0^ [54]. However, the production cost of PCU is higher. The source of B present in the PCU + Ur-NBPT + NI + B + S mixture was identified as ulexite, an evaporite formed under arid conditions in saline lakes supported by hydrothermal vents and linked to volcanic activity [56]. For this reason, ulexite may be associated with other evaporites, such as halite, gypsum, glauberite, and bassanite, as described in Figure 3h.

### 4.2. Ammonia Volatilization of N Sources in Clayey and Sandy Soil

The losses by NH_3_-N volatilization were higher in sandy soil for all N sources tested (Figure 4 and Figure 5). This behavior occurred due to the higher moisture content of the sandy soil, resulting from the higher rainfall volume 24 h before the application of the nitrogen fertilizers as a topdressing (Figure 1). Under dry soil conditions, the urease hydrolysis rate is low; however, the rate increases as the soil water content increases [57]. Above 20% moisture, hydrolysis is practically no longer affected by changes in soil moisture [4].

The previous considerations explain the sigmoidal behavior of NH_3_-N volatilization losses, which depend on the increase in urease enzyme activity [58], which consumes the H^+^ resulting from urea hydrolysis, as demonstrated by the reaction CO(NH_2_)_2_ + 2H^+^ + 2H_2_O → 2NH_4_^+^ + H_2_CO_3_ [59]. This reaction promotes the increase in soil pH around N fertilizer granules to approximately 8.7, changing the balance between NH_4_^+^ and NH_3_ [60]. After reaching the maximum loss (α), NH_3_-N emissions decrease over time, due to the gradual reduction in pH and stabilization of N in the form of NH_4_^+^-N [24]. 

In addition to differences in soil moisture, the clay content and, consequently, the cation exchange capacity (CEC) (Table 1) are the main differences between the two soil classes studied that will also influence the intensity of NH_3_-N volatilization. Clayey soils with a higher CEC have more exchange sites to retain the NH_4_^+^ produced in the hydrolysis of urea due to adsorption. In addition, the higher buffering capacity of soils with a higher CEC provides greater resistance to changes in soil pH around N granules caused by the urease enzyme, thus decreasing the intensity of NH_3_ -N volatilization [58,59]. Therefore, the lower loss of NH_3_-N by volatilization in clayey soils resulted in higher inflection points in the NH_3_-N curve (β) and a lower MDL, as described in Table 2 and Table 3.

Ammonium sulfate and ammonium nitrate + calcium sulfate had the lowest accumulated losses of NH_3_-N in relation to the other N sources in both production environments (Figure 4), due to the absence of N in the amidic form (NH_2_-N). Corrêa et al., Minato et al., and Otto et al. [13,14,24] also obtained low losses due to NH_3_-N volatilization with the use of N sources in the ammoniacal and nitric forms, with losses ranging from 0.7 to 5.2% and 1.0 to 7.7% of the N applied for ammonium sulfate and ammonium nitrate, respectively, depending on the dose of applied topdressing. Following the increasing order of NH_3_-N emissions, Ur-formaldehyde was the enhanced-efficiency source that most reduced NH_3_-N volatilization, but it was less efficient than ammoniacal and nitric sources. Although Ur-formaldehyde can reduce the solubility of N fractions through the synthesis of methylene urea groups, it contains some urea that does not react with formaldehyde (Figure 3e), favoring losses by volatilization of NH_3_-N, even in small proportions.

The next formulation considered is Ur-NBPT + Duromide, a stabilizer that combines two NBPT + Duromide molecules, both of which inhibit the activity of the urease enzyme, but having the advantage of a more stable chemical structure under low pH and high soil temperature conditions [9]. However, the use of Ur-NBPT + Duromide showed higher emissions of NH_3_-N (mean of 23.5% of the applied N) when compared to meta-analysis studies that obtained losses by volatilization of NH_3_-N of 14.8% using NBPT [26]. Under conditions with large amounts of straw on the soil surface, such as those described in the present study (mean of 3.95 Mg ha^−1^ Brachiaria straw), the amount of urease enzyme in the soil will be higher, increasing losses by NH_3_-N volatilization by up to 25.5% [7]. In other words, the stabilizers can reduce, but not eliminate, the activity of the urease enzyme, possibly due to the high amounts of this enzyme in systems with high amounts of straw. Thus, such materials are not the most appropriate technology for production environments with large amounts of straw residues on the soil surface.

The mixture of PCU + Ur-NBPT + NI was inefficient in reducing losses by NH_3_-N volatilization, with losses very close to those of conventional urea (Figure 4 and Table 2). As PCU + Ur-NBPT + NI is a mixed fertilizer, composed of different granules, the granules were separated to understand the efficiency of each component in reducing or contributing to NH_3_-N emissions (Figure 5). Although PCU granules are designed to release N at a controlled rate to synchronize with the crop demand and reduce environmental pollution by NO_3_^−^-N, NH_3_-N and N_2_O-N [61], factors such as high temperatures, excessive rainfall, the number and thickness of the coating layers, and quality of the coating material may have interfered with the efficiency of PCU, contributing to the release of N in the amidic form and losses in the form of NH_3_-N.

On the other hand, the addition of NI to Ur-NBPT to mitigate direct emissions of N_2_O-N [28] and losses by leaching of NO_3_^−^-N [62] was the factor that most contributed to the inefficiency of the mixture PCU + Ur-NBPT + NI, since the addition of NI to NBPT significantly decreased the ability of NBPT to inhibit urea hydrolysis by up to 21% [63], in addition to contributing to the increase in NH_3_-N volatilization losses [30,31].

### 4.3. Nitrification Inhibitor Increases Losses Due to Ammonia Volatilization

The results showed that the use of NI significantly increased the volatilization of NH_3_-N relative to the PCU granules, especially in sandy soil (Figure 5b). According to Wu et al. [30], there are two main mechanisms associated with increased volatilization: (i) NIs are a group of chemical compounds that inhibit the activity of *Nitrosomonas* spp. bacteria responsible for the oxidation of NH_4_^+^ to nitrite (NO_2_^−^) and, therefore, increase the soil concentration of NH_4_^+^ that is converted to NH_3_; and (ii) NIs induce a liming effect. Qiao et al. [64] found that the application of NI increased soil pH by 0.23 units, due to the decelerated rate of nitrification and increased efficiency of N use by plants resulting from the lower leaching of NO_3_^−^. Thus, unleached NO_3_^−^ is absorbed by plant roots, which excrete OH^−^ to maintain the electrochemical balance in the soil, thus increasing the pH of the medium [64,65]. Once the soil pH changes, the balance between NH_3_ and NH_4_^+^ is affected; as the soil pH increases, the equilibrium shifts, leading to the transformation of NH_3_-N and its subsequent loss to the atmosphere in the form of gas [60].

The volatilization of NH_3_-N can be influenced by several factors, such as dose, N source, climatic conditions, management system, and soil attributes, with the latter being the main factor responsible for altering the efficiency of NIs. For example, a meta-analysis performed by Kim et al. [66] found that treatments with NI increased emissions of NH_3_-N in soils with higher pH (5.4 to 7.9) and smaller ranges of CEC (5.7 to 16.8 cmol_c_ dm^−3^) in comparison with lower-pH soils (4.7 to 6.2) and larger CEC ranges (10.0 to 24.0 cmol_c_ dm^−3^). This effect occurs due to the favored formation of NH_3_-N at basic pH, combined with soils of low CEC, which provide fewer exchange sites for NH_4_^+^ adsorption, facilitating the loss of N by volatilization [67].

Another important soil attribute is the organic matter (OM) content. Soils with high levels of OM have higher amounts of *Nitrosomonas* spp., which hinders the performance of NIs [29] and leads to a need for higher concentrations of NI in soils with high OM content. In addition, high clay contents will favor lower emissions of NH_3_-N, due to their contribution to increasing soil CEC [68]. This explains the higher volatilization of urea treated with NI in sandy soil, since the pH, OM, CEC, and clay content were 4.50 and 5.70, 2.89 and 1.42%, 11.06 and 4.19 cmol_c_ dm^−3^, and 78 and 10% for the clayey and sandy soils, respectively (Table 1). Therefore, the use of NIs, especially in sandy textured soils in rainfed agriculture, is not recommended as a strategy to increase the NUE. The INs technology in nitrogen fertilizers is more efficient in flooded agriculture systems, due to the denitrification losses of N_2_O-N and N_2_, representing up to 34% of the applied N [69]. 

### 4.4. Nitrogen Sources and Nutrient Concentration of Corn—Macronutrients

Although there were differences in losses due to NH_3_-N volatilization between N sources, the N applied as topdressing fertilization that was not lost by volatilization may have been sufficient to meet the N demand of the corn crop. As a result, changes in the concentration N status were not observed between the N sources, but only in relation to the control that did not receive N as topdressing (Figure 6a). According to Cantarella et al. [4], in many cases, most of the N absorbed by crops comes from soil OM, and fertilizer N, although important to increase yield, provides complementary N. Similarly, Oliveira et al. [70], working with ^15^N isotopes, observed that only 33% of the N absorbed by corn plants was derived from topdressing nitrogen fertilization.

Topdressing N fertilization was important for the maintenance of leaf chlorophyll content, which was indirectly quantified by the increase in SPAD index in relation to the control in both production environments (Figure 7f). According to Taiz et al. [3], chlorophylls are green photosynthetic pigments that have a porphyrin-like ring structure with a Mg atom coordinated in the center, linked to four other N atoms, with a long tail of hydrocarbons. Thus, in the absence of N, the plant degrades chlorophyll molecules to obtain the four N atoms that are part of its structure, developing generalized chlorosis in the leaf and compromising the absorption of light.

The highest leaf N concentration and chlorophyll content (SPAD) being in the corn grown in clayey soil was due to the higher expected yield provided by the better fertility of clayey soils than sandy soils, requiring a higher photosynthetic rate [71], and the higher N uptake by the plants, since each ton of corn grains requires 21.5 kg of N [35]. This effect was not observed in the accumulation of leaf N among the controls, most likely due to the lack of fertilization as topdressing, which inhibited the realization of the productive potential of the clayey soil environment.

After N, K is the nutrient most absorbed by corn plants, followed by P. Thus, the increase in cultivation intensity and, thus, higher yields obtained through N fertilization provided greater absorption of K and P and consequently a higher accumulation of these nutrients in the leaf (Figure 6b,c). Since P is a key element for the synthesis of molecules such as DNA, RNA, ATP, and NADPH [72], and as K is important for the activation of enzymatic systems and protein synthesis [73], synergistic interactions exist between N × P and N × K [74]. For example, Rietra et al. [75] performed a meta-analysis on the interaction between nutrients and found a synergism between N × P and N × K and no case of antagonism, in a total of 77 studies. The highest leaf concentration of P being in corn grown in sandy soil resulted from a lower adsorption to iron and aluminum oxides, which has a positive correlation with clay content [76]. Higher concentrations of leaf K were also observed in corn grown in sandy soil due to the low CEC of the soil, providing lower adsorption of K^+^ and consequently greater availability in the soil solution.

Elements are absorbed at different rates, due to their affinity with membrane carriers, obeying the decreasing cationic order NH_4_^+^ > K^+^ > Na^+^ > Mg^2+^ > Ca^2+^ [38]. Thus, because Ca is absorbed by the roots in the form of Ca^2+^, its absorption may be compromised by the high concentrations of NH_4_^+^ in the soil solution, due to competition [77]. Therefore, the highest accumulation of leaf Ca being in the control cultivated in clayey soil, relative to the values obtained with ammonium sulfate, ammonium nitrate + calcium sulfate, and PCU + Ur-NBPT + NI (Figure 6d), occurred due to two factors: (i) high doses of fertilizers containing NH_4_^+^; and (ii) the use of fertilizers with NIs. The use of NIs will inhibit the nitrification process, increasing the level of NH_4_^+^ in the soil, thus suppressing the absorption of Ca^2+^ and resulting in a lower accumulation in leaves. This effect was also observed in the reduction in leaf Mg levels in corn grown in clayey soil using ammonium sulfate compared to the control (Figure 6e). Thus, high concentrations of NH_4_^+^ can also reduce the absorption of Mg^2+^ and K^+^ by plants [78,79,80].

However, unlike Ca, the use of ammonium nitrate + calcium sulfate promoted an increase in leaf Mg, due to the presence of dolomite in its composition (Figure 3c), which inhibits the undesirable exothermic decomposition process of ammonium nitrate [49], in addition to being an important source of Mg^2+^ to plants [81]. On the other hand, reductions in leaf K accumulation were not observed with the use of ammoniacal sources, because the suppression is greater with divalent cations (Ca^2+^ and Mg^2+^) than with monovalent cations (K^+^) [82]. Regarding the differences between the production environments, the control conducted in sandy soil obtained lower concentrations of leaf Mg because the soil content was below the critical level (Table 1), which was 1 cmol_c_ dm^−3^ [35]. For ammonium nitrate + calcium sulfate, the highest concentration of leaf Mg in corn grown in clayey soil resulted from the highest dose of nitrogen application in topsoil, which thus provided more Mg in the form of dolomite.

Many plant compounds, such as amino acids and proteins, have both N and S, which helps explain the existence of a positive N/S ratio and the increases in leaf S concentration in both corn production environments with N application as topdressing compared to the control (Figure 6f). However, ammonium sulfate was the N source that provided the highest leaf S concentrations, due to the high concentration of S per unit mass (24%) in the form of sulfate (SO_4_^2−^), which is the main form absorbed by plants and does not require oxidation by *Thiobacillus*, which, in turn, is dependent on soil temperature and moisture conditions [83]. On the other hand, the sandy soil probably favored more intense leaching of SO_4_^2−^ due to the few anionic adsorption sites, decreasing the contact with the root system and, consequently, absorption by the plant [84]. This mechanism explains the higher concentration of S in the leaves of corn grown in clayey soil, as described in Figure 6f.

### 4.5. Nitrogen Sources and Nutrient Concentration of Corn—Micronutrients

The nitrogen fertilizers did not alter the leaf concentrations of Fe and Mn. However, the leaf concentrations varied between the production environments. The natural levels of micronutrients in soil depend on the chemical composition of the parent material, pedogenetic processes, and the degree of soil weathering [85]. Thus, the parent material of the clayey soil located in northern Paraná state is basalt, an igneous rock rich in micronutrients such as Fe and Mn, because these elements have the same geochemical formation environments [86]. In contrast, the sandy soil located in the northwestern part of the Paraná state originates from sandstones of the Caiuá Formation, a sedimentary rock whose main constituent mineral is quartz [87]. Consequently, higher levels of Fe and Mn are naturally available in clayey soil, favoring greater absorption and concentration of these nutrients in corn leaves (Figure 7a,b).

The use of nitrogen fertilizers in agriculture promotes the production of H^+^ through the oxidation of ammonium to nitrate, as demonstrated by the nitrification reaction NH_4_^+^ + 2O_2_ → NO_3_^−^ + H_2_O + 2H^+^ [88]. With a reduction in soil pH, the availability of metal cationic micronutrients increases [38], providing higher leaf concentrations of Zn in corn grown in sandy soil (Figure 7c) and Cu in corn grown in clayey soil (Figure 7d). However, the highest concentrations of leaf Zn occurred with the application of ammonium sulfate, which is an exclusively ammoniacal source, and the soil acidification process was intensified because more NH_4_^+^ was provided as a substrate for nitrifying bacteria [89]. An increase in leaf Zn with the use of ammonium sulfate was not observed in clayey soil, due to the higher CEC and OM content, which favored the acidity buffering reaction through the exchange of H^+^ ions for basic cations (Ca^2+^, Mg^2+^, K^+^ and Na^+^) in clay minerals and OM [90], which is the predominant buffering mechanism in the pH range between 4.2 and 5.0 [91].

Regarding the differences in Zn levels between the production environments, the higher grain yield obtained in clayey soil (Figure 8a) required a higher Zn absorption by the plants, because Zn is the most exported micronutrient in corn, with 24.8 g for each ton of grain produced [35]. Cooper, in turn, is the micronutrient that interacts the most with soil organic compounds and forms stable complexes, especially with carboxylic and phenolic groups of OM, in addition to having a strong affinity for clay [92]. Therefore, sandy soils, with low OM levels, are mostly deficient in Cu, due to leaching losses. This explains the higher concentration of leaf Cu in clayey soils than in sandy soils (Figure 7d).

The presence of ulexite in the PCU + Ur-NBPT + NI mixture identified by XRD (Figure 3h) resulted in an increase in leaf B concentration in both corn production environments (Figure 7e). However, soluble B sources such as borax (Na_2_B_4_O_7_·10H_2_O) and boric acid (H_3_BO_3_) are more commonly used to maintain plant growth when compared to lower-solubility sources such as ulexite (NaCaB_5_O_9_·8H_2_O) and colemanite (Ca_2_B_6_O_11_·5H_2_O) [93]. However, the ulexite present in the PCU + Ur-NBPT + NI mixture is of acid origin and is obtained through the granulation process with the use of sulfuric acid, which provides an increase in water solubility of approximately 90%. This higher solubility favors the faster release of B, making the nutrient available for plant absorption and increasing leaf B concentrations.

### 4.6. Clay Soils Are More Responsive to Nitrogen Fertilization

The sandy soil, because it originated from the Caiuá sandstone formation, is characterized by low a CEC, due to its high sand content, especially coarse sand [87]. A low CEC directly affects cation losses, due to leaching, and consequently the expected crop yield. The low fertility of sandy soils was confirmed by the lower growth of corn plants in the sandy soil than in the clayey soil (Figure 8b). Thus, in a production environment with a low response to fertilization, topdressing nitrogen fertilization contributes less to increases in corn yield (Figure 8a). In a meta-analysis performed by Tremblay et al. [94], the authors concluded that corn yield increased by a factor of 1.6 in sandy soils and 2.7 in clayey soils after nitrogen fertilization, showing that corn is more responsive to N fertilization in clay soils.

In addition to the influence of CEC on the response to nitrogen fertilization, soil texture can play a role. For example, clay affects the stabilization of organic N through the protection of OM by aggregates, favoring the preservation of microbial biomass [95]. Ros et al. [96] studied the variation in mineralizable N and its relationship with physical properties in 98 agricultural soils in the Netherlands and observed a lower N mineralization rate in clayey soils than in sandy soils. Ping, Ferguson, and Dobermann [97] found that corn needed less N fertilizer in sandy soils than in clayey soils. This may suggest that soil texture influences the degree of OM stabilization and, consequently, the response to nitrogen fertilization, increasing the chances of an increased yield via N fertilization in clayey soils. 

Soil texture also provides different degrees of water storage in the soil. Therefore, sandy soils, due to their higher porosity, store less water, resulting in higher metabolic costs to the plant to absorb water and promote transpiration, which can consequently affect the yield. Although the response to nitrogen fertilization is lower in sandy soils than soils with other textures, studies of fertilization with varying sources and doses of N in these locations should be performed, mainly because such soils are highly susceptible to losses by NH_3_-N volatilization and because they are the main soils of new agricultural frontiers in Brazil [98,99].

The lower losses by NH_3_-N volatilization obtained with the use of ammonium sulfate and ammonium nitrate + calcium sulfate increased the corn yield in clayey soil. With a reduction in NH_3_-N losses, N will be used more efficiently by the plant, favoring the synthesis of biomolecules essential for corn growth and development. However, other characteristics of these N sources may also have influenced the yield and should be mentioned. For example, the higher concentration of leaf S provided by the application of ammonium sulfate may have contributed to the increase in corn yield, because S is closely linked to N metabolism, converting nonprotein N into protein [83]. Thus, all plant metabolism depends on S compounds, due to the structural functions they perform, such as maintaining the active conformation of proteins through the disulfide bonds between methionine and cysteine (S-S) and metabolic functions, since they constitute amino acids, coenzymes, and proteins with Fe and S [3,100].

Not only did ammonium nitrate + calcium sulfate generate a low loss by NH_3_-N volatilization, but synergistic benefits for plant growth have been observed if NO_3_^-^ and NH_4_^+^ are provided together [80,101]. The beneficial effect of the simultaneous supply of these two inorganic forms of N occurs due to the lower suppression of the absorption of cationic nutrients, mainly Ca^2+^ and Mg^2+^, by the exclusive supply of NH_4_^+^ [80], lower acidification or alkalinization of the rhizosphere as a result of the absorption in excess of NH_4_^+^ or NO_3_^−^ [65], and lower energy requirements for NH_4_^+^ assimilation than NO_3_^−^ assimilation, given that NO_3_^−^ cannot be used directly by plants until it is reduced to NH_4_^+^; a reduction catalyzed sequentially by nitrate reductase enzymes and nitrite reductase [102,103].

The N source PCU + Ur -NBPT + NI also increased the corn yield in clayey soil. However, this effect was not caused by a reduction in NH_3_-N volatilization, but by the supply of B via ulexite and synchronization of N release with PCU. Boron is responsible for plant functions such as sugar translocation and the regulation of carbohydrate and phytohormone metabolism. However, B plays a very important role in the metabolism of N. This is due to the requirement of B for the synthesis of the uracil nitrogen base, an essential component of RNA, which is also indispensable for ribosome formation and protein synthesis [38]. Therefore, an increased availability of B in soil favors higher yields, mainly because it is a nutrient found in low concentrations in tropical and subtropical soils, due to losses by leaching in the form of H_3_BO_3_^0^.

Even given the inefficiency of PCU granules in reducing losses by NH_3_-N volatilization (Figure 5a), in most cases, controlled-release technology can decrease the availability of N at the beginning of corn development, when the absorption is still low [27], and increases the availability of N in phenological stages VT (tasseling) to R1, when the demand for N by corn is high [104]. This behavior in the later release stages of N was observed through the maximum daily loss of NH_3_-N in PCU granules alone (β), which occurred 30.6 days after topdressing fertilization in the clayey soil (Table 3); with enough time for the corn to be in VT, which usually coincides with the eighth week after emergence [105].

## 5. Conclusions

The losses by NH_3_-N volatilization were up to 46% of the N applied with urea. However, NI addition to urea increased N losses by NH_3_-N volatilization by 8.8 and 23.3% in relation to only urea for clayey and sandy soils, respectively. This leads to important implications for the use of NI as a mitigation tool for climate change in rainfed agriculture. 

The nitrogen fertilizer technologies applied in a topdressing on clayey and sandy soil presented the following, in decreasing order, losses by volatilization of NH_3_-N: urea > URP + Ur-NBPT + IN > Ur-NBPT + Duromide > Ur-formaldehyde > ammonium nitrate + calcium sulfate > ammonium sulfate. 

Soil with a clayey texture was 38.4% more responsive to nitrogen fertilization than soil with a sandy texture. The increase in corn grain yield in the clayey soil did not occur only due to the reduction in losses by NH_3_-N volatilization, other factors, such as S and B supplementation and N release at a controlled rate, to synchronize with the crop demand, also influenced the increase in corn yield. However, it is always advisable to choose N sources that increase crop yield, while generating the lowest possible losses due to NH_3_-N volatilization. In this study, these sources were ammonium sulfate and ammonium nitrate + calcium sulfate, which contributed to reductions in NH_3_-N emissions of 84 and 80% in relation to urea, respectively, thus favoring more profitable and sustainable agriculture. 

## Figures and Tables

**Table 1:** Chemical and granulometric analysis of an Oxisol (clayey texture), Ultisol (sand texture) and interpretation of values for the surface layer (0.00–0.20 m).

Soil Properties	Clayey Soil ^1^	Sand Soil ^2^	Soil Attribute
	0.00–0.20 m	0.00–0.20 m	Interpretation ^3^
pH CaCl_2_	4.50	5.70	Medium ^1^/High ^2^
H + Al (cmol_c_ dm^−3^)	6.49	1.90	-
Al^3+^ (cmol_c_ dm^−3^)	0.15	0.00	Very low ^1,2^
Ca^2+^ (cmol_c_ dm^−3^)	3.31	1.68	High^1^/Medium ^2^
Mg^2+^ (cmol_c_ dm^−3^)	1.09	0.52	Medium ^1,2^
K^+^ (cmol_c_ dm^−3^)	0.17	0.09	Medium ^1^/Low ^2^
SB (cmol_c_ dm^−3^)	4.57	2.29	-
CEC_pH7_ (cmol_c_ dm^−3^)	11.06	4.19	Medium^1^/Very low ^2^
ECEC (cmol_c_ dm^−3^)	4.72	2.29	High^1^/Medium ^2^
BS (%)	42	55	Medium^1^/High ^2^
P (mg dm^−3^)	16.15	56.61	Very high ^1,2^
S (mg dm^−3^)	2.96	0.95	Medium ^1^/Very low ^2^
B (mg dm^−3^)	0.40	0.12	High ^1^/Low ^2^
Zn (mg dm^−3^)	5.70	12.96	High ^1^/Very high ^2^
Cu (mg dm^−3^)	10.32	5.04	Very high ^1,2^
Fe (mg dm^−3^)	49.14	25.86	-
Mn (mg dm^−3^)	142.26	47.28	Vey high ^1^/high ^2^
OC (g dm^−3^)	16.73	8.26	High ^1^/Medium ^2^
OM (%)	2.89	1.42	High ^1^/Low ^2^
Sand (%)	8	89	-
Silt (%)	14	1	-
Clay (%)	78	10	-

pH(CaCl_2_) (0.01 mol L^−1^) at a soil:solution ratio of 1:2.5; H + Al was determined by the Shoemaker–McLean–Pratt (SMP) method; Ca^2+^, Mg^2+^, and Al^3+^ extracted with KCl 1 mol L^−1^; OM: soil organic matter content obtained by organic carbon × 1.724 (Walkley-Black); P, K^+^, Zn, Cu, Fe, and Mn: Mehlich-1 extraction; SO_4_^2−^ was extracted by calcium phosphate in acetic acid; B was extracted with hot water; sum of bases (SB): Ca^2+^ + Mg^2+^ + K^+^; CEC: cation exchange capacity at pH 7 (SB + H + Al); ECEC: effective cation exchange capacity (SB + Al^3+^); BS: base saturation [(SB/CEC) × 100]; and particle size distribution (sand, silt, and clay) by densimeter method. ^1^ Soil attribute interpretation of the clayey soil. ^2^ Soil attribute interpretation of the sand soil. ^3^ Interpretation of soil attributes, according to SBCS/NEPAR [35].

**Table 2:** Nonlinear regression parameters adjusted (logistic model) for NH_3_-N volatilization cumulative losses for conventional and enhanced efficiency nitrogen fertilizers and reduction of NH_3_-N emission in relation to urea for clayey and sandy soils.

Treatments	Soil and Rate at Topdressing	Parameters				MDL	Reduction of Losses of NH_3_-N in Relation to Urea (%)
		α	γ	β	R^2^	kg ha^−1^ day^−1^ NH_3_-N	
		kg ha^−1^ NH_3_-N		day			
Urea		41.0	3.3	8.4	0.98	3.10	-
Amm. sulfate		8.5	3.8	8.3	0.97	0.56	79.3
Amm. nitrate + Cal. sulfate	Clayey	10.7	5.0	7.3	0.96	0.54	73.9
Ur-NBPT + Duromide	(200 kg ha^−1^)	33.4	3.1	8.5	0.98	2.69	18.5
Ur-formaldehyde		18.3	3.2	6.0	0.96	1.43	55.4
PCU + Ur-NBPT + NI		40.1	5.5	11.1	0.96	1.82	2.2
Urea		69.2	2.5	1.2	0.97	6.92	-
Amm. sulfate		11.0	5.8	7.1	0.98	0.47	84.1
Amm. nitrate + Cal. sulfate	Sandy	13.6	5.3	3.5	0.95	0.64	80.3
Ur-NBPT + Duromide	(150 kg ha^−1^)	44.5	1.8	3.6	0.97	6.18	35.7
Ur-formaldehyde		30.5	2.7	1.6	0.97	2.82	55.9
PCU + Ur-NBPT + NI		62.5	4.7	2.2	0.93	3.32	9.7

α: maximum cumulative volatilization; β: time at which 50% of the losses occur, corresponding to the curve inflection point; γ: parameter of the equation used to calculate the MDL (maximum daily loss of NH_3_-N).

**Table 3:** Nonlinear regression parameters adjusted (logistic model) for NH_3_-N volatilization cumulative losses of the separate granules PCU and Ur-NBPT + NI and the increase of NH_3_-N emission in relation to urea for clayey and sandy soils.

Treatments	Soil and Rate at Topdressing	Parameters				MDL	Increased of Losses of NH_3_-N in Relation to Urea (%)
		α	γ	β	R^2^	kg ha^−1^ day^−1^ NH_3_-N	
		kg ha^-1^ NH_3_-N		day			
PCU	Clayey	40.3	11.3	30.6	0.98	0.89	-
Ur-NBPT + NI	(200 kg ha^−1^)	44.6	3.8	9.0	0.98	2.93	8.8
PCU	Sandy	44.9	11.5	12.6	0.97	0.98	-
Ur-NBPT + NI	(150 kg ha^−1^)	85.3	2.5	0.7	0.97	8.53	23.3

α: maximum cumulative volatilization; β: time at which 50% of the losses occur, corresponding to the curve inflection point; γ: parameter of the equation used to calculate the MDL (maximum daily loss of NH_3_-N).

## Data Availability

The data presented in the manuscript are the sole data, and no other data are linked with this data.
